# Role of employee loneliness, job uncertainty and psychological distress in employee-based brand equity: Mediating role of employee exhaustion

**DOI:** 10.3389/fpubh.2022.941106

**Published:** 2022-10-07

**Authors:** Hao Chen, Jingya Li, Juan Li, Jiaying Bao

**Affiliations:** ^1^School of Economics and Management, Wuhan University, Wuhan, China; ^2^School of Economics and Management, Ningxia University, Yinchuan, China; ^3^School of Education and Professional Development, University of Huddersfield, Huddersfield, United Kingdom; ^4^School of Literature and Media Institute, Baise University, Baise, China

**Keywords:** job uncertainty, psychological distress, emotional exhaustion, employee health, brand equity

## Abstract

Employee-based brand equity plays a crucial role in building organizations' brand equity, and organizations strive to maintain it because of its stimulating effect on competitive achievement. Based on psychological contract and stress theory, this study developed a model that points out the antecedents which can play an adverse role in the EBBE building process. This study explores the role of employee loneliness, job uncertainty, and psychological distress on employee-based brand equity. This study also explores the mediating role of emotional exhaustion in these relationships. For the empirical analyses of the model, this study gathered data based on a 459 sample size under a time-lag approach from the employees of clothing brands in China. This study analyzed the data through partial least square structural equation modeling (PLS-SEM). For this purpose, SmartPLS software was used. The outcomes revealed that employee loneliness has no direct relationship with employee-based brand equity; however, job uncertainty and psychological distress negatively influence employee-based brand equity, such as job uncertainty and psychological distress reduce employee-brand-based equity. Moreover, emotional exhaustion mediates the relationship between employee loneliness and employee-based brand equity and job uncertainty and employee-based brand equity; however, emotional exhaustion does not mediate the relationship between psychological distress and employee-based brand equity. Finally, practical implications, limitations, and future directions are discussed in this study.

## Introduction

In this turbulent environment, organizations seek ways to deal with the market dynamics to maintain their sustainability (1). Organizations put their efforts into strengthening tangible and intangible assets to differentiate themselves in the market. However, Piehler et al. (2) revealed that firms are now giving more importance to intangible assets (human capital specifically) for gaining a competitive edge. Human capital could play a considerable role and assist firms in dealing with the challenges of the turbulent environment of markets. Scholars also draw firms' attention to consider the constructive role of human capital in the creation of brand equity and in enhancing the firm's overall performance (3–5).

Hanaysha and Al-Shaikh (6) stated that brand equity indicates the value of a firm's product or services due to its unique brand name or attributes. Further, they acknowledged that firms gain fruitful paybacks of brand equity through higher profits and long-term market survival. King and Grace (7) revealed that there is plenty of knowledge in literature about brand equity from financial and customer perspectives. However, Erkmen (8) noticed that the building of brand equity is ignored in literature from employees' perspectives. Employees are a valuable part of organizations, and they can play a significant role in making or breaking the brand. Poulis and Wisker (9) quantified the employees as the firm's asset and commented that organizations can use intellectual abilities of employees as strategic weapon to build brand equity. Boukis and Christodoulides (4) acknowledged that employee-based brand equity (EBBE) is a valuable asset for an organization. Further, they commented that creating and maintaining EBBE is a crucial task for organizations. Yang et al. (10) revealed that the COVID-19 pandemic effects deeply affect the emotional and psychological health of employees. Moreover, employees' overall brand performance is also influenced by psychological and emotional discomfort at the workplace.

Poulis and Wisker (9) stated that the EBBE building process positively influences when they feel a psychological attachment with their organizations. However, according to Jindo et al. (11) point of view, organizations have to keep a deep eye on the psychological health of employees and cure them from being a victim of psychological distress in the workplace. Moreover, they acknowledged that employee psychological distress negatively affected their brand performance. Takao et al. (12) also informed about the negative consequences of employee psychological distress and said that it is an alarming situation for organizations when their employees experience psychological distress in the workplace. Moreover, employees' job outcomes also decrease when they feel stress, anxiety, and uncertainty in the workplace.

Chen and Eyoun (13) point out that with unexpected experience of COVID-19 lockdown, employees feel insecure about their employment. Employees are feeling restless about their future and have a fear of job loss. Moreover, this stress and uncertainty about job unfavorably impact their workplace performance and productivity. Scholars identified job uncertainty as a determinant of decreasing job satisfaction and commitment and a damaging tool for the psychological health of employees (14, 15). Bazzoli and Probst (16) said that employee job uncertainty could also negatively influence the cognitive and critical abilities of employees. Further, Chen and Eyoun (13) revealed that employee job uncertainty has a crucial role in enhancing their emotional exhaustion.

According to Wong et al. (17) point of view, employee emotional exhaustion is a crucial factor for firms. Further, they argue that employee emotional exhaustion can create undesirable circumstances for a firm's brand equity and sustainability. Consequently, EBBE also influences negatively when employees experience emotional exhaustion at the workplace. Employee emotional exhaustion paves the way for negative consequences for organizations in decreasing employees' job performance, lowering their work effectiveness, and increasing turnover intentions (18). Further, they informed that organizations should have to focus on building effective strategies and preventive measures to cope with employees' emotional exhaustion state. Seppala and King (19) revealed that emotional exhaustion and burnout of employees not only effects on work performance of employees but also take them into a lonely state.

Peng et al. (20) define loneliness as an employee's state of mind when they feel emotionally exhausted and isolate themselves from other human beings. Further, they stated that workplace loneliness is a damaging association with employee wellbeing and job performance. Moreover, it also damages the ability of employees' creativity and critical thinking capacity. Yang and Wen (21) noticed that employee socialization with the leadership and other team members could play a crucial role in mitigating the negative impact of workplace loneliness on employees' behavior. Further, they acknowledged that employee loneliness also negatively influences their brand-related performance.

The current study serves the literature in different ways. First, the present study extends the literature by providing insight into employee-based brand equity. Based on the psychological contract theory (22) and stress theory (23), the present study tries to attempt the role of negative employee attributes (employee loneliness, emotional exhaustion, psychological distress) on EBBE. According to the author's knowledge, this is the only study that provides insights to firms on EBBE. Second, this study also points out three negative attributes of the workplace that can cause the reduction of EBBE. Third, the present paper attempts to find the association between employee loneliness and EBBE. Fourth, finding out the relationship between emotional exhaustion and EBBE is also an objective of the present paper. Fifth, this study also tries to check the relationship between psychological distress and EBBE. Lastly, this study attempts to determine the mediating role of employee emotional exhaustion between independent variables employee loneliness, emotional exhaustion, psychological distress, and dependent variable EBBE, respectively.

## Literature review

### Employee-based brand equity

Prados-Pena and Del Barrio-García (24) defines brand equity as value addition activities of firms to strengthen the company's position through differentiating their offering products or services. King and Grace (25) highlighted that financial, customer and employee are three approaches for measuring the firm's brand equity. Further, they explained these approaches and said that the financial-based brand equity approach could be defined as the additional economic worth in the form of cash flows. Consumer-based brand equity is a measure that indicates the perceptions, feelings, and affiliations of consumers toward the brand. Lee et al. (26) define employee-based brand equity as the employees' constructive brand behaviors and efforts in building a firm's brand equity.

Poulis and Wisker (9) identified that “brand endorsement, brand-consistent behaviors, and brand allegiance” are three essential dimensions of employee-based brand equity. Further, they elaborate brand endorsement as the degree to which employees willingly endorse the brand to their internal and external stakeholders with positive word of mouth. Brand-consistent behaviors could be termed employees' workplace behaviors that represent the firm's values and norms (3). The third key dimension is the brand alliance, which demonstrates the employees' attention and planning to be a part of that firm for a long time. Additionally, King and So (27) stated that these three dimensions of employee-based brand equity are a valuable indicator of organizational equity and its long-term sustainability.

Anasori et al. (28) noticed that employees' psychological engagement has an essential role in the brand-building process of firms. In addition, employees' job performance and productivity are also influenced positively when they have an emotional and psychological bond with organizations. However, Bentley et al. (29) noticed that psychological stress or anxiety at the workplace could cause a reduction in commitment and trust of employees that, in turn, decrease their overall performance. Weak employment relationships between employees and organizations could be a hazardous situation for firms' sustainability. Employees also feel uncertain about their jobs and future when their psychological and emotional needs don't fulfill at the workplace (30). Said and Tanova (31) notify that these negative attributes may cause employees' emotional exhaustion. In addition, emotionally exhausted employees may come into a dangerous psychological state known as loneliness. Due to loneliness, the employee-based brand equity process could influence negatively because employees don't have an interest in the internal value creation activities of the firm.

## Employee loneliness

Workplace loneliness is an apathetic psychological state that refers to the individuals' set of feelings when they perceive that their social needs are not sufficiently met by their peers and organization (32). Ayazlar and Güzel (33) point out two important distinctions in the literature regarding loneliness definition. First, social loneliness indicates the dearth of social relationships or acceptable friendship relations. The second one is emotional loneliness which refers to the absence of affective commitment or romantic relationships. Further, they commented that loneliness has painful cognitive attitudinal, behavioral, and emotional outcomes for employees, which influences severely on their workplace performance. Peng et al. (20) also noticed that workplace loneliness negatively affects employees' wellbeing and job performance. Further, they point out that workplace loneliness makes emotional and psychological changes in employees that negatively affect employees' creativity ability.

Konno et al. (34) revealed that during the COVID-19 pandemic, workplace loneliness severely influenced employees' mental health. Indeed, the workforce who experience loneliness during the epidemic may feel psychologically disengaged and incompetent for performing work activities. Moreover, employees' self-efficacy and work performance also decrease, which is a turbulent situation for firms' sustainability. Additionally, Kloutsiniotis et al. (35) quantified workplace loneliness as a “modern epidemic” that needs to be cured. Further, they informed that the firms should take proactive measures to prevent their workforce from victimizing loneliness. Ayazlar and Güzel (33) points out “emotional deprivation and social companionship” as two important dimensions of workplace loneliness. Emotional deprivation could be defined as the extent to which employees have an interpersonal relationship within the workplace. Social companionship is the extent to which employees have an adequate social circle in the workplace. Employees share their knowledge and problems with their peers when they have a strong social network.

Konno et al. (34) point out that workplace loneliness paves the way for employees' psychological disorders, such as anxiety, depression, and psychological distress. Burris et al. (36) also stated that psychologically disengaged employees might not have a strong bond with the values and objectives of the firm. Consequently, employees' brand-related performance may also influence negatively, which is an alarming situation for organizations. Based on the stress theory, the present study assumes that employee-based brand equity influences negatively when they experience loneliness in the workplace. For empirical investigation, present study hypothesize that

***H1:*** *Employee loneliness has a negative association with employee-based brand equity*.

### Job uncertainty

Bordia et al. (37) defines uncertainty as the extent to which individuals are unable to predict something accurately. In other words, uncertainty could be termed as a sense of doubt and insecurity about upcoming events. Further, they commented on uncertainty and said this type of situation might occur due to disinformation and ambiguous or contradictory information. Chen and Eyoun (13) acknowledged that job uncertainty includes employees' ambiguity or doubts about their long-term employment relationship with the organization. Additionally, Bordia et al. (37) highlighted four types of taxonomies of uncertainty. First, external uncertainty could be termed as the environmental uncertainty that occurs due to technological and market changes. Second, organizational uncertainty could occur due to changes in the external business environment. Third, group uncertainty arises due to changes in internal strategies and structure within the firm. Fourth, individual uncertainty occurs when employees feel insecure regarding their job role and status in organizations.

Vu et al. (15) noticed that quantitative job insecurity and qualitative job insecurity are two important types of job securities. Quantitative type refers to employees; perceived threat of job loss in future, and qualitative insecurity indicates the perceived threat of impairing the quality of employment relationship such as lack of trust lessening development opportunities. According to Ravn and Sterk (14) point of view, job uncertainty has several adverse outcomes for employees' wellbeing in the form of stress, mental disorders and emotional exhaustion. Further, they identified that employees' job satisfaction, commitment and trust in the organization are negatively influenced, lowering their productivity.

Han et al. (38) noticed that employees' job uncertainty increased after the COVID-19 pandemic. Further, they stated that job uncertainty severely threatens employees' mental and emotional health, lowering their job performance. Chen and Eyoun (13) identified that job uncertainty of employees adversely influences the psychological health of employees. Further, they argue that job-related uncertainties trigger employees' emotional health, and they feel emotionally exhausted at the workplace. Bazzoli and Probst (16) also point out negative consequences of job uncertainty and said that it might trigger employees' cognitive processes which in turn is an alarming situation for firms. Further, they argue that employees' job insecurity at the workplace also influences their brand-related performance. Vu et al. (15) also identified that employees' job insecurity is a threatening situation for a firm's effectiveness.

Based on the above-discussed literature, the present study attempts to reveal how job uncertainty influences the brand-related performance of the employees. With the support of psychological contract theory (22), the present study assumes that when employees perceive that their jobs are not secured, and they feel uncertainty about losing a job at the workplace, their brand-related performance is also influenced negatively. For empirical investigation, the present study hypothesizes that:

***H2:*** *Job uncertainty has a negative relationship with employee-based brand equity*.

## Psychological distress

Bentley et al. (29) acknowledged that psychological distress at the workplace has negative consequences on employees' mental wellbeing. Further, they defined psychological distress and said that it is an emotional suffering state of an individual which is associated with stress and tensions. Moreover, they stated that it is very difficult for employees to cope with these stressors in routine life. Anasori et al. (28) identified that psychological distress adversely affects on attitudes and behaviors of employees. Dunleavy et al. (30) revealed that psychological distress at the workplace might cause severe types of mental health disorders in employees. Further, they point out that employees' mental health matters a lot for their efficient performance in the workplace. Moreover, organizations may bear high costs of employees' psychological distress in decreasing working outcomes (29). Therefore, organizations must realize the importance of employees' mental health to enhance their overall performance. Dunleavy et al. (30) shed further light and said the workforce's mental health is an upcoming challenge for firms. Additionally, firms have to focus on coping strategies and policies that can address mental health issues appropriately.

Scholars reported that, during the pandemic of COVID-19, employees feel isolated due to preventative measures of COVID-19 (34, 39). These isolated lives adversely impact employees' mental health, and consequently, their psychological distress levels increases. Additionally, employees' work outcomes are negatively influenced by psychological distress, and they feel emotionally exhausted at the workplace (29). However, Anasori et al. (28) acknowledged that the psychological distress of employees not only impacts emotionally but also damages their critical abilities. Moreover, they revealed that psychological distress also paves the way to heighten employee turnover intentions. According to Bashir et al. (40), when employees feel psychological disengaged and dissatisfied at the workplace, the chances of their psychological contract breach are greater than before. Poulis and Wisker (9) also commented that the psychological disengagement of employees could pave the way for damaging the brand equity performance of employees.

Based on stress theory, the present study assumes that when employees feel psychological distress at the workplace, their work outcomes are adversely influenced by this emotional suffering. Additionally, employees' brand-building efforts are also influenced unfavorably when they show negative behaviors in the workplace. Based on the above-discussed literature, the present study hypothesizes that:

***H3:*** *Psychological distress has a negative relationship with employee-based brand equity*.

## Emotional exhaustion

Said and Tanova (31) define emotional exhaustion as the extent to which individuals feel emotionally worn out due to accumulated stress from the workplace and personal lives. Further, they acknowledged that emotional exhaustion is a mental depletion state, which often causes poor workforce performance and depletes organizational e?ectiveness. Chen et al. (41) identified that the literature on emotional exhaustion divided it into physical and psychological stresses. Further, they stated that both these types severely affect the productivity of employees and organizations. According to Said and Tanova (31), disproportionate job demands or stress may be the crucial cause of employee emotional exhaustion. Loh and Saleh (42) noticed that employees' emotional exhaustion increased their withdrawal behaviors.

Employees' burnout at the workplace is an important factor in their emotional exhaustion (19). Moreover, employees' mental and emotional health is adversely influenced by their emotional exhaustion, which lowers their work productivity. Chen and Eyoun (13) noticed that the COVID-19 pandemic also has some adverse consequences on the psychological, behavioral, and emotional health of employees. Further, they stated that employees' emotional exhaustion is highly reported during the peak days of the epidemic. Further, Said and Tanova (31) commented on the consequences of emotional exhaustion and said that it has severe outcomes in the form of employee performance reduction and decreasing the firm's productivity. More broadly, employees' emotional exhaustion negatively influences service delivery capability and brand-related performance. Wong et al. (17) also stated that employees' emotional exhaustion harm employees' brand-building efforts.

**Figure 1:**
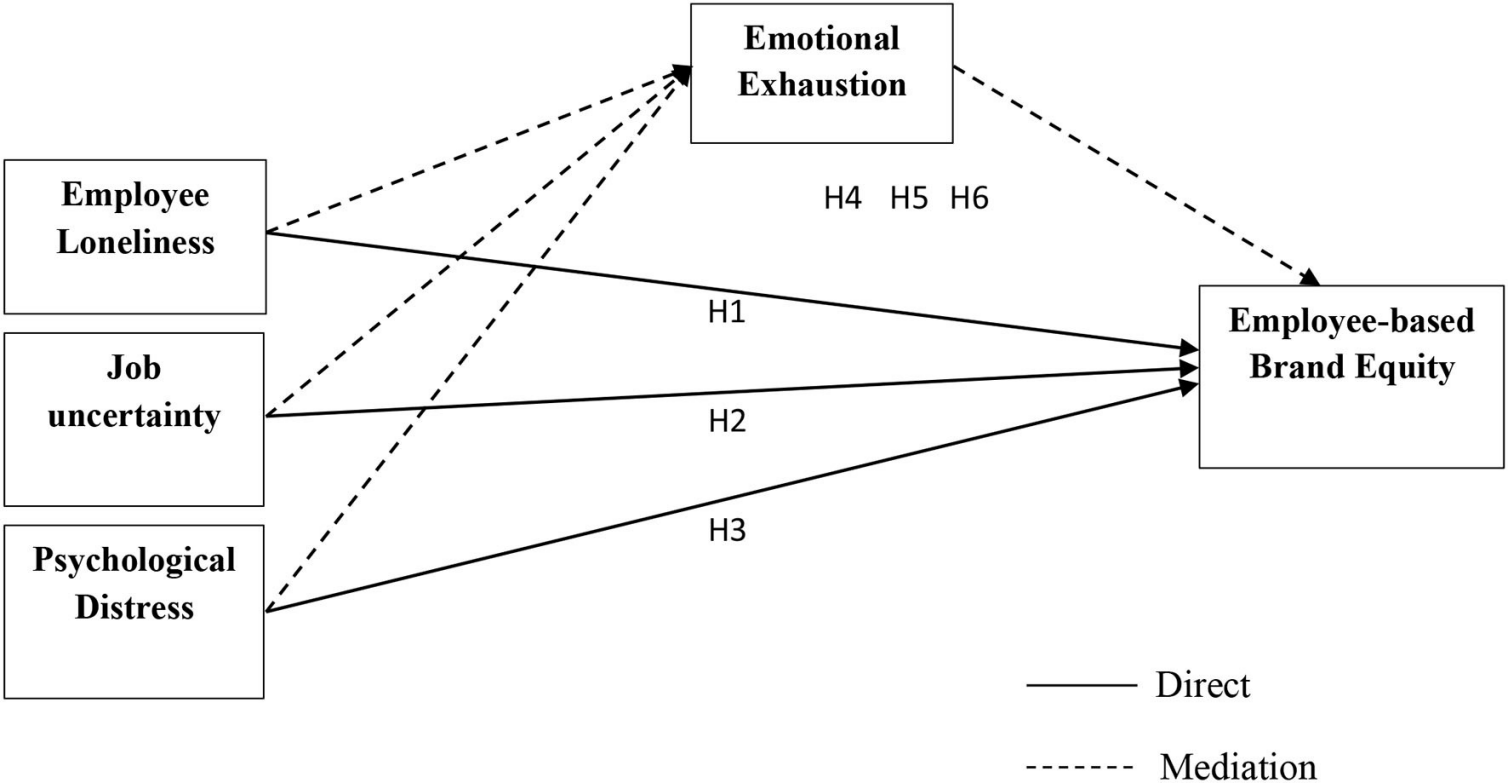
Conceptual framework.

Wong et al. (17) point out that employees feel emotionally exhausted when they experience loneliness at the workplace. In addition, employees' emotional and psychological health is influenced adversely, and they become the victim of mental disorders. Konno et al. (34) added the vein and said that psychological distress is one of the crucial psychological states that cause a decrease in employees' workplace performance. With the support of literature present study assumes the following hypotheses, and Figure 1 represents this study model.

***H4:*** *Employee emotional exhaustion mediates the relationship between employee loneliness and employee-based brand equity*.
***H5:*** *Employee emotional exhaustion mediates the relationship between job uncertainty and employee-based brand equity*.
***H6:*** *Employee emotional exhaustion mediates the relationship between psychological distress and employee-based brand equity*.

## Research methods

### Study design

A convenient sampling method was followed to collect the data for this study from the various employees of clothing brands operating in China. To finalize the targeted clothing brands, the author interviewed different groups of students to know the perception of the people or community about the famous clothing brands. The author asked the students about clothing brands and based on their opinions; the author finalized the most repeated brands that were famous among them. The author also consulted with senior researchers, and after their opinions, the author finalized targeted brands. The author contacted managers of targeted clothing brands and convinced them to a meeting, and upon their consent, the author fixed a meeting with them. The author personally visited and did a brief meeting with them about the objective of this study research, and after their satisfaction, as the online data collection would be more convenient, the author asked the managers to add their employees to a WeChat group for data collection. The author also assured them that the managerial implication would be shared with them at their request after completing the research. Finally, managers showed their consent. First, the author shared a cover letter that briefed the employees about their data confidentiality and trusted them that the data would be used for academic objectives and aggregated outcomes would be revealed as individual-level responses would be destroyed. In this way, employees agreed to participate in this research activity. After that, the author shared the link of the WeChat group with managers, and managers shared links with their employees, and those employees that had consent added themselves to that group. In this way, the responses were gathered without any pressure but with employees' consent.

Moreover, the cover letter also assured the employees that no answer is right or wrong; their true answer would be considered right for this study's natural outcomes; hence avoid consultation with your colleague during answering. This step surely boosted their confidence, and they filled the questionnaire with their natural responses. The author developed the electronic questionnaire in the google form and translated it into Chinese for employees' better understanding. The senior researchers also verified the translated questionnaires and sample-based data also gathered on these questionnaires for language improvement. This study also adopted the time lag approach for data gathering and collected data in different turns to reduce common method bias. However, the author included a hidden code in the questionnaire to recognize the same respondents in all turns. The author gathered data on demographic information and independent variables (employee loneliness, job uncertainty, and psychological distress) in the first turn and on the dependent variable (employee-based brand equity) in the second turn. The author collected data on the mediator variable (emotional exhaustion) in the third turn. In the first turn, the author collected 755 responses. In the second turn, the author collected 513 questionnaires, and in the third turn, the author collected 459 responses. Finally, the outcomes of this study were developed based on the 459 sample size. The respondents' demographic information is shown in Appendix 2 in Supplementary material.

### Measures

This study examined the participants' responses on five points Likert scale. This scale consists of 1 to 5 numbers, 1 represents “strongly disagree,” 2 represents “disagree,” 3 represents “neutral,” 4 represents “agree,” and 5 represents “strongly agree.” This study measured variables based on previously validated items. The construct of employee loneliness was measured with ten items scale developed by Russel (43) and used by Garg and Anand (44). The sample item included “do you feel a lack of companionship?” The construct of job uncertainty was measured with five items scale developed by Bordia et al. (45) and used by Paulsen et al. (46). The sample item included, “Very soon existing policies and procedures will change.” The construct of psychological distress was measured with three items scale developed by Barnett and Brennan (47) and used by Lapalme et al. (48). The sample item included, “I feel worried and anxious.” The construct of emotional exhaustion was measured with seven items scale developed by Paulsen et al. (46). The sample item included, “I feel fatigued when I get up in the morning and have to face another day on the job.” The employee-based brand equity construct was measured with five items scale developed by Boukis and Christodoulides (4). The sample item includes “I don't consider the impact on the company's brand when I make decisions.”

## Results

### Assessment of measurement and structural model

The results of this study were assessed by using the partial least square structural equation modeling (PLS-SEM) technique. The PLS-SEM is a variance-based technique unrelated to the covariance-based technique (49). The PLS-SEM is selected because it is equally suitable for confirmatory and exploratory studies (50). Structural equation modeling (SEM) is based on two techniques such as covariance-based structural equation modeling (CB-SEM) and partial least square structural equation modeling (PLS-SEM). The PLS-SEM is useful for advancing and extending the theory, whereas CB-SEM is useful for accepting and rejecting the theory (50). PLS-SEM is also effective for small data size analysis as it efficiently handles it. Therefore, this study assessed model outcomes by applying the PLS-SEM technique through Smart PLS software. The PLS-SEM examines data in two steps. In the first step, it examines the measurement of the model, and in the second step, it assesses the structural path.

The measurement outcomes of this study are based on two different parts: first, it measures model reliability, and second, it examines model validity. Cronbach alpha, roh-A, composite reliability, and average variance extract (AVE) were considered for the reliability assessment of this study model (50, 51). Table 1 depicts this study's model reliability. First, according to the threshold, the Cronbach alpha and composite reliability values should be >0.7 (49). Out study model variables have Cronbach alpha and composite reliability values >0.7; for instance, this study's independent variables Employee loneliness, job uncertainty, and psychological distress and mediator variable emotional exhaustion and dependent variable employee-based brand equity Cronbach alpha values are 0.917, 0.855, 0.833, 0.914 and 0.868 and composite reliabilities are 0.930, 0.896, 0.900, 0.932 and 0.901 respectively are >0.7 hence Cronbach values and composite reliability values are accepted. Similarly, the roh-A values are also according to the given threshold. Moreover, according to the criteria, the AVE values of variables should be >0.5. In this study model, all variable values are >0.5. Hence AVE values of all variables are also accepted (52). The present study data model constructs graphical representation of Cronbach alpha, composite reliability, roh-A, AVE and R^2^ are shown in Appendix 1 in Supplementary material.

**Table 1:** Reliability and convergent validity of the study constructs.

**Construct**	**Item**	**Outer loadings**	**VIF**	**Alpha**	**roh-A**	**Composite reliability**	**AVE**
EBBE	EBBE1	0.817	2.577	0.868	0.889	0.901	0.647
	EBBE2	0.784	3.234				
	EBBE3	0.790	3.297				
	EBBE4	0.804	2.130				
	EBBE5	0.825	2.227				
EEX	EEX1	0.710	1.604	0.914	0.914	0.932	0.662
	EEX2	0.816	2.432				
	EEX3	0.848	2.915				
	EEX4	0.866	3.079				
	EEX5	0.835	2.581				
	EEX6	0.849	2.788				
	EEX7	0.759	2.005				
EL	EL1	0.751	2.117	0.917	0.924	0.930	0.571
	EL2	0.706	2.357				
	EL3	0.768	1.878				
	EL4	0.822	2.280				
	EL5	0.800	3.076				
	EL6	0.776	2.721				
	EL7	0.712	2.247				
	EL8	0.728	2.177				
	EL9	0.713	2.502				
	EL10	0.772	2.104				
JU	JU1	0.851	2.257	0.855	0.860	0.896	0.634
	JU2	0.737	1.719				
	JU3	0.795	1.853				
	JU4	0.789	1.842				
	JU5	0.804	1.838				
PD	PD1	0.825	1.736	0.833	0.846	0.900	0.750
	PD2	0.876	2.067				
	PD3	0.895	2.107				

EBBE, employee based brand equity; EEX, emotional exhaustion; EL, employee loneliness; JU, job uncertainty; PD, psychological distress.

**Figure 2:**
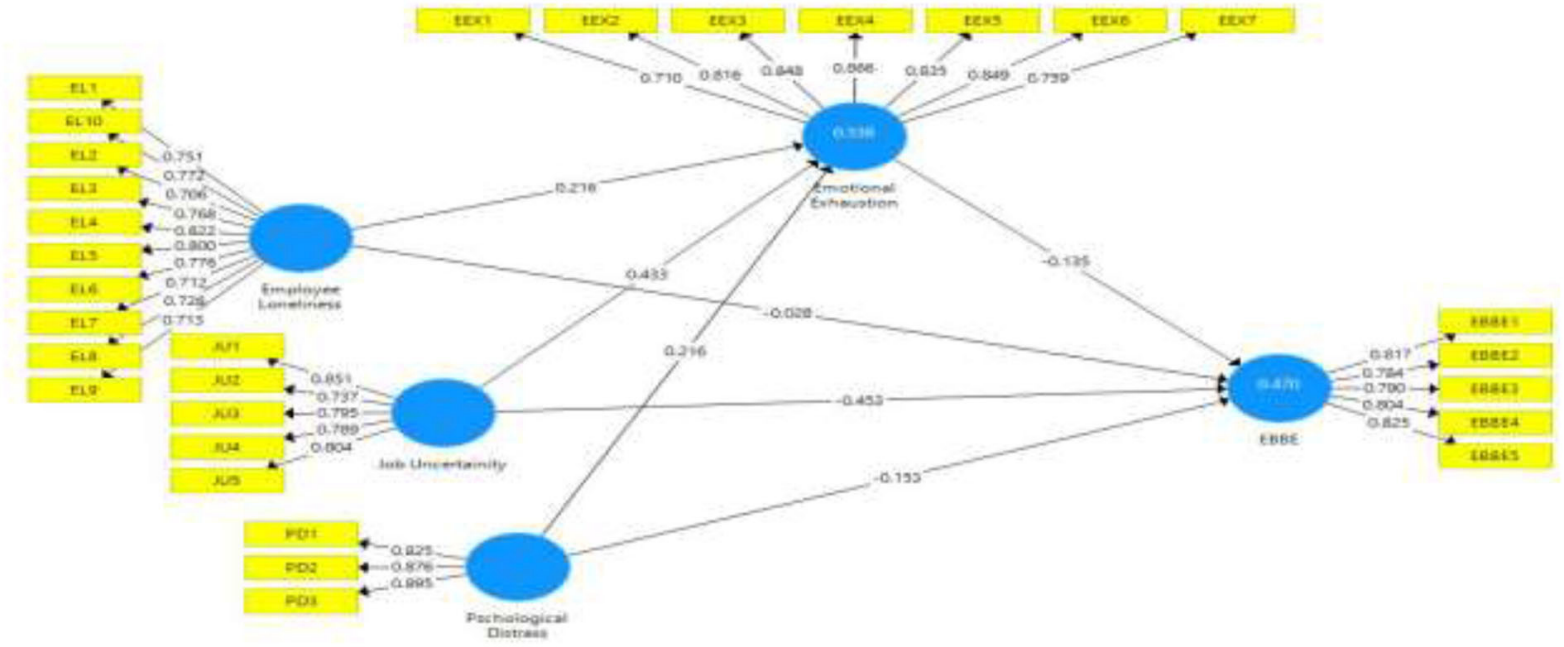
Path estimates.

Table 1 also explains the factor items' outer loading. According to the threshold, a value >0.7 is considered appropriate for the model (50). In our model, all variables (employee loneliness, job uncertainty, psychological distress, emotional exhaustion, and EBBE) items values are >0.7 (Figure 2). Hence all values are accepted. Moreover, Table 1 also represents variance inflation factor (VIF) values of all present study model construct items. VIF is examined to identify the collinearity issue in the model. According to the criteria, a value below 0.5 is considered appropriate because it is considered without collinearity (49). The present study model constructs item EBBE3 shows the highest VIF value (3.297) compared to other items. Hence, the outcomes revealed that the model of the present study is free from collinearity issues.

The R^2^ values of the latent construct explain the model strength, such as a value up to 0.5 shows moderate strength and >0.5 shows substantial strength (49). Our model latent constructs emotional exhaustion and EBBE R^2^ values are 0.538 and 0.470, respectively, showing the substantial and moderate strength of the model. Hence, the model R^2^ values showed 53.8% variance in emotional exhaustion and 47.0% variance in EBBE. The latent constructs values Q^2^ greater than zero are considered appropriate for the model. Our study model latent variables have greater than zero Q^2^ values. Hence, it shows that model of this study is significant.

The discriminant validity of our model was measured through two well-known approaches, such as Fornell-Larcker and heterotrait-monotrait (HTMT) criteria (50). The Fornell-Larcker criterion is measured by taking the square roots of AVE values of all constructs' (51, 53). Table 2 represents that this study constructs Forenell-Larcker values. According to the threshold, all columns in the table should have an above value greater than their below values. The outcomes of this study are according to the Fornell-Larkcer given threshold as the above values are shown in bold in Table 2 are greater than their below values. Thus, discriminant validity is confirmed in the present study model. Moreover, according to the criteria, the HTMT values of all constructs should be <0.85 but must be 0.9 (49, 52). According to the outcomes shown in Table 3, our model constructs values are <0.85. Hence, this study model HTMT discriminant validity is also achieved.

**Table 2:** Discriminant validity (Fornell-Larker-1981 criteria).

**Construct**	**EBBE**	**EEX**	**EL**	**JU**	**PD**
EBBE	**0.804**				
EEX	−0.551	**0.814**			
EL	−0.370	0.511	**0.756**		
JU	−0.662	0.678	0.442	**0.796**	
PD	−0.561	0.618	0.479	0.688	**0.866**

EBBE, employee based brand equity; EEX, emotional exhaustion; EL, employee loneliness; JU, job uncertainty; PD, psychological distress.

**Table 3:** Discriminant validity (HTMT).

**Construct**	**EBBE**	**EEX**	**EL**	**JU**	**PD**
EBBE	–	**–**	**–**	**–**	**–**
EEX	0.601	–	**–**	**–**	**–**
EL	0.399	0.537	–	–	–
JU	0.726	0.760	0.492	–	–
PD	0.620	0.702	0.552	0.810	–

EBBE, employee based brand equity; EEX, emotional exhaustion; EL, employee loneliness; JU, job uncertainty; PD, psychological distress.

### Hypotheses testing

This study statistics outcome for hypotheses empirical analysis was conducted through 5,000 samples of bootstrapping approach. Table 4 depicts the present study's direct, indirect and total path results (50, 51). This study considered the *t* and *p* values of statistics outcomes to accept and reject the hypotheses (51). The hypotheses outcomes of the present study are presented in Table 5. H1 of this study proposed that employee loneliness negatively impacts EBBE. The statistics results (t = 0.613, *p* = 0.540) revealed that employee loneliness does not directly influence the EBBE. Hence, H1 of the present study is rejected. H2 of the present study proposed that job uncertainty negatively influences EBBE. The statistics outcome (t = 6.912, p = 0.000) has confirmed that job uncertainty influences the EBBE. According to the beta value, it is also confirmed that job uncertainty negatively influences the EBBE; for instance, one unit change in job uncertainty brings a −0.453 negative change in EBBE, such as job uncertainty reduces the EBBE. Hence, H2 is accepted. H3 of this study proposed that psychological distress negatively influences the EBBE. The statistics outcomes of this study (*t* = 2.594, *p* =0.010) have confirmed that psychological distress influences the EBBE, and the beta value confirmed that psychological distress negatively influences the EBBE. For example, one unit change in psychological distress leads to −0.153 negative change in EBBE. Hence, H3 is accepted.

**Table 4:** Direct, indirect and total path estimates.

**Direct path**	**Beta**	**SD**	**t**	**Confidence interval (95%)**	**f^2^ Effect size**	***p***
EEX → EBBE	−0.135	0.060	2.238	(0.019 to 0.259)	0.016	**0.025**
EL → EBBE	−0.028	0.045	0.613	(−0.062 to 0.117)	0.001	**0.540**
EL → EEX	0.216	0.045	4.837	(0.135 to 0.310)	0.075	**0.000**
JU → EBBE	−0.453	0.065	6.912	(0.319 to 0.578)	0.163	**0.000**
JU → EEX	0.433	0.045	9.620	(0.344 to 0.520)	0.207	**0.000**
PD → EBBE	−0.153	0.059	2.594	(0.035 to 0.274)	0.021	**0.010**
PD → EEX	0.216	0.049	4.452	(0.120 to 0.311)	0.049	**0.000**
**Indirect path**					
EL → EEX → EBBE	−0.029	0.014	2.025	(0.004 to 0.061)	**0.043**
JU → EEX → EBBE	−0.059	0.027	2.157	(0.008 to 0.117)	**0.031**
PD → EEX → EBBE	−0.029	0.015	1.927	(0.004 to 0.063)	**0.054**
Total Path					
EEX → EBBE	−0.135	0.060	2.238	(0.019 to 0.259)	**0.025**
EL → EBBE	−0.057	0.044	1.300	(−0.029 to 0.143)	**0.193**
EL → EEX	0.216	0.045	4.837	(0.135 to 0.310)	**0.000**
JU → EBBE	−0.511	0.060	8.541	(0.389 to 0.624)	**0.000**
JU → EEX	0.433	0.045	9.620	(0.344 to 0.520)	**0.000**
PD → EBBE	−0.182	0.059	3.089	(0.070 to 0.302)	**0.002**
PD → EEX	0.216	0.049	4.452	(0.120 to 0.311)	**0.000**

EBBE, employee based brand equity; EEX, emotional exhaustion; EL, employee loneliness; JU, job uncertainty; PD, psychological distress.

**Table 5:** Hypotheses testing.

	**Hypotheses**	**Coefficient (Beta)**	**S.D**	***t***	**Confidence interval (95%)**	**f^2^ Effect size**	***p***	**Status**
H1	EL → EBBE	−0.028	0.045	0.613	(−0.062 to 0.117)	0.001	0.540	Not supported
H2	JU → EBBE	−0.453	0.065	6.912	(0.319 to 0.578)	0.163	0.000	Supported
H3	PD → EBBE	−0.153	0.059	2.594	(0.035 to 0.274)	0.021	0.010	Supported
**Mediation hypotheses**						
H4	EL → EEX → EBBE	−0.029	0.014	2.025	(0.004 to 0.061)	0.043	Supported
H5	JU → EEX → EBBE	−0.059	0.027	2.157	(0.008 to 0.117)	0.031	Supported
H6	PD → EEX → EBBE	−0.029	0.015	1.927	(0.004 to 0.063)	0.054	Not supported

EBBE, employee based brand equity; EEX, emotional exhaustion; EL, employee loneliness; JU, job uncertainty; PD, psychological distress.

This study also seeks the mediation effect of emotional exhaustion in the relationship between employee loneliness and EBBE, job uncertainty and EBBE, and psychological distress and EBBE, respectively. The H4 of the present study proposed that the relationship between employee loneliness and EBBE is mediated by employee exhaustion. The statistics outcome of H4 (*t* = 2.025, *p* = 0.043) revealed that employee loneliness mediates the negative relationship between employee loneliness and EBBE, such as EBBE is reduced through employee loneliness mediation. Hence, H4 is accepted. H5 of this study proposed that the relationship between job uncertainty and EBBE is mediated by emotional exhaustion. According to the statistics outcomes (*t* = 2.157, *p* = 0.031), it has been confirmed that emotional exhaustion mediates the negative relationship between job uncertainty and EBBE, such as the mediation effect of emotional exhaustion reduced the EBBE. Hence, H5 is accepted. H6 of this study proposed that the association between psychological distress and EBBE mediates by EBBE. The statistics outcomes (*t* = 1.927, *p* = 0.054) have confirmed that emotional exhaustion does not mediate the relationship between psychological distress and EBBE. Hence, H6 is rejected.

## Discussion

Due to environmental turbulence, organizations' worries have increased to overcome market dynamics about maintaining sustainability (1). EBBE paves the way to developing a competitive edge for organizations. Hence, previous studies have emphasized the ways of building EBBE (4, 26, 54) but ignored exploring the factors that decrease employees-brand building behavior. Hence, this study developed a model by exploring the various employees' organizational-based factors that intentionally reduce employee-brand building behavior. This study explored how emotional loneliness, job uncertainty, and psychological distress influence the EBBE under the psychological contract and stress theory (23, 55). This study found that employee loneliness did not influence EBBE directly (Table 5). According to Yang and Wen (21), socialization between leader and employee or between the leader and other team members can reduce the employee's negative effect on their organizational behavior. For instance, employees socially feel a high bond and connection that would positively reduce their stress and negative emotion. Hence, this hypothesis outcomes are not found and thus rejected. This study found that job uncertainty has a negative effect on EBBE. Such as job uncertainty directly reduces the employee-based brand equity. The study by Chen and Eyoun (13) revealed that COVID-19 lockdown is an incredible experience, and employees feel uncertain outcomes regarding their job. Hence, employees negatively speak out about their organization under such circumstances, which is against their brand-building behavior. The outcomes regarding the relationship between psychological distress and EBBE revealed that psychological distress negatively impacts EBBE, such as psychological distress decreasing the EBBE. The outcomes of this study are consistent with the findings of prior studies (12, 34). These studies point out that the work productivity of employees decreases when they feel psychological and emotional distress at workplace.

Moreover, this study also assumes emotional exhaustion as the mediator in the relationship between employee loneliness and EBBE, psychological distress and EBBE and job uncertainty and EBBE, respectively. According to the outcomes, emotional exhaustion mediates the negative relationship between employee loneliness and EBBE and job uncertainty and EBBE, respectively. These findings have consistency with prior studies (17, 34, 56). According to these studies, the employees feel emotionally exhsuated when they experience loneliness and distress at workplace, and in turn their brand-building effoerts also reduces. According to outcomes, the relationship between psychological distress and EBBE does not mediate by emotional exhaustion. However, these results are not consistent with prior studies (17, 34). According to these studies, employees suffer from mental diseases as a result of the unfavorable effects on their emotional and psychological health. Konno et al. (34) stated that psychological distress is one of the key psychological conditions that contribute to a decline in employees' performance at work.

## Theoretical and practical implications

This study contributed to the literature in several ways. First, this study extends the literature on EBBE by exploring factors (employee loneliness, job uncertainty, and psychological distress) that negatively affect the building process of EBBE. This study points out the three possible antecedents that lead the EBBE building process in negative directions. This study also provides guidelines to managers and firms on how they can improve the EBBE building process by dealing with these three negative attributes of employees (employee loneliness, job uncertainty, and psychological distress). Moreover, this study strengthens the key role of such negative factors on EBBE under the support of psychological contract theory and stress theory. Based on stress theory, the present study serves the literature by shedding light on employee loneliness as an antecedent to reducing the EBBE building process. In addition, with the support of psychological contract theory, this study assumes that employees' uncertainty adversely affects their trust level, and they feel like their expectations are not met by their organizations. EBBE building process adversely affects over time when the trust level of employees decreases and they feel insecure about their jobs. This study also serves the literature by adding the evidence on stress theory from the perspective of psychological distress and EBBE. The present study assumes that when employees feel psychological distress at the workplace, their work outcomes are adversely influenced by this emotional suffering. In return, the brand-building efforts of employees are also influenced unfavorably. In addition, this study extends the literature on emotional exhaustion as the mediator in the relationship between employee loneliness and EBBE and job uncertainty and EBBE.

Practically this study provides guidelines to firms that the negative attributes of employees (employee loneliness, psychological distress, and job uncertainty) could be possible antecedents that can play an adverse role in organizational sustainability and brand-building activities. Therefore, the organization should build a supportive culture in the shape of top management support and leadership support for employees. This support may not eliminate but surely reduce the feeling of employee loneliness, psychological distress, and job uncertainty.

## Limitations and future research directions

Like other social studies, this study also has limitations. These limitations can be proposed to extend this study. First, this study gathered data through a questionnaire survey method. The sample is also small. So future research can consider other data collection methods and enlarge the sample size to validate the present study outcomes. Second, this study only considered clothing brand employees for data collection; hence future research should collect data from other brands and compare the outcomes with our study. Third, this study considered emotional exhaustion as a mediator; however, the results revealed that it did not mediate the relationship between psychological distress and EBBE, so future research has the opportunity to add other mediators like demotivation and employee cynicism to investigate the findings of the model further. Fourth, this study did not check the moderating effect in this model, so future research may consider some moderators like emotional intelligence and self-efficacy to extend the results of the present study. Finally, this study is conducted in China, and the results are generalizable for the Chinese context only; future studies may expand the scope to other regions to enhance the reliability and generalizability of the results.

## Conclusion

In this competitive era, EBBE facilitates organizations to achieve a competitive advantage. However, the organizations are striving to maintain EBBE. Under the support of psychological contract and stress theory, this study develops a model to recognize those factors that decrease the EBBE. This study found that job uncertainty and psychological distress negatively influence EBBE. The direct impact of employee loneliness on EBBE was not found in this study. Moreover, this study found that the relationship between employee loneliness and EBBE and psychological distress and EBBE was mediated by emotional exhaustion; EBBE did not mediate the relationship between psychological distress and EBBE. This study guides that organizations must develop a supportive culture that can play a vital role in reducing employees' loneliness, job uncertainty and psychological distress because these negative factors decrease employee-based brand equity. Under these factors, employee exhaustion also increased, which mediated these relationships to reduce EBBE.

## Data availability statement

The original contributions presented in the study are included in the article/supplementary material, further inquiries can be directed to the corresponding author/s.

## Ethics statement

The studies involving human participants were reviewed and approved by Lanzhou City University, China. The patients/participants provided their written informed consent to participate in this study. The study was conducted in accordance with the Declaration of Helsinki.

## Author contributions

JiL conceived. HC designed the concept. JuL collected the data and wrote the paper. JB read and agreed to the published version of the manuscript. All authors contributed to the article and approved the submitted version.

## Conflict of interest

The authors declare that the research was conducted in the absence of any commercial or financial relationships that could be construed as a potential conflict of interest.

## Publisher's note

All claims expressed in this article are solely those of the authors and do not necessarily represent those of their affiliated organizations, or those of the publisher, the editors and the reviewers. Any product that may be evaluated in this article, or claim that may be made by its manufacturer, is not guaranteed or endorsed by the publisher.
