# Supplementary material for: Role of employee loneliness, job uncertainty and psychological distress in employee-based brand equity: Mediating role of employee exhaustion
Source: Front Public Health. 2022 Oct 7;10:941106. doi: 10.3389/fpubh.2022.941106 (PMC9585936; doi:10.3389/fpubh.2022.941106)
Supplement: Supplementary file 1 [file Data_Sheet_1.docx]

**APPENDIX-1**

**
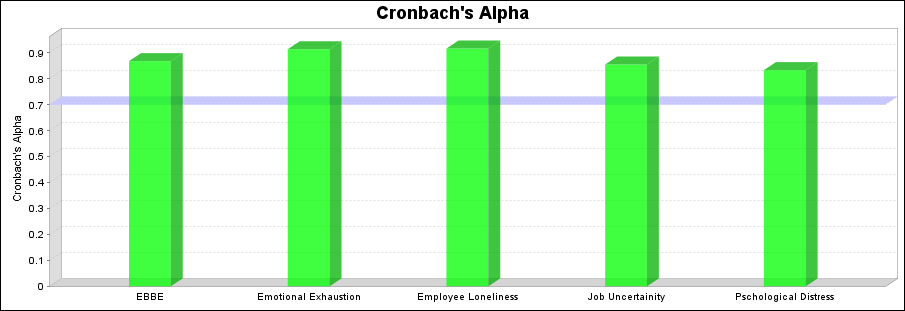
**


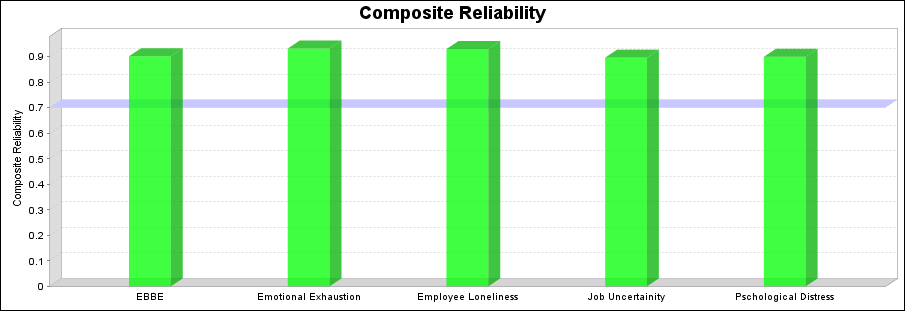


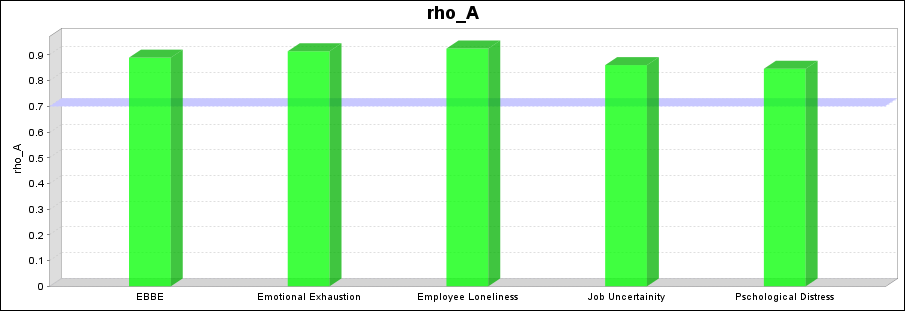


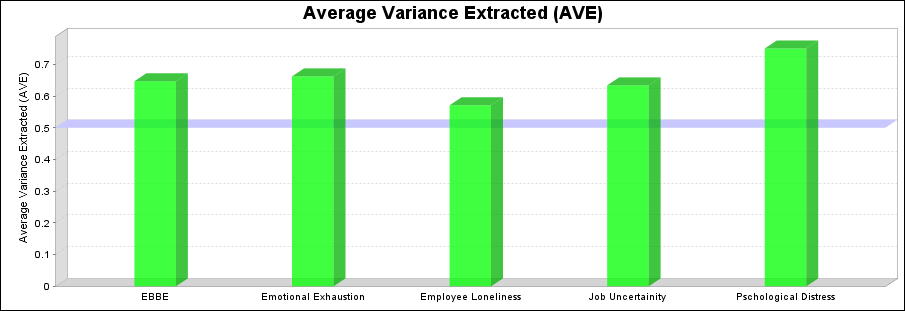


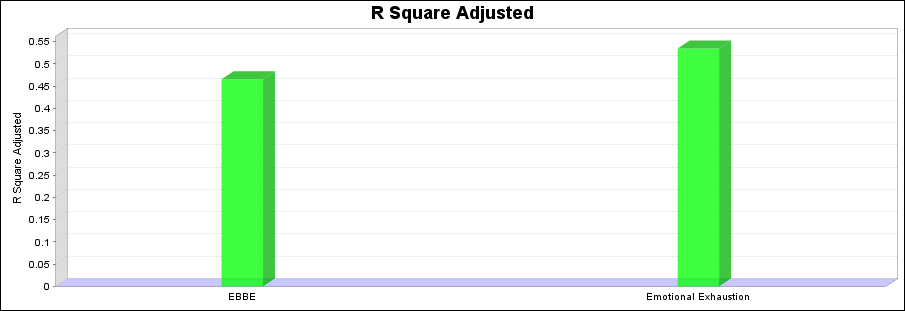


**APPENDIX-2**

**Demographic Information**

| **Categories** | **Subcategories** | **Numbers** | **Percentage** |
| --- | --- | --- | --- |
| Gender | Male | 263 | 57.3 |
|  | Female | 196 | 42.7 |
| Age | 26-33 years | 107 | 23.3 |
|  | 33-40 years | 157 | 34.3 |
|  | 40-47 years | 118 | 25.7 |
|  | 48 years or above | 77 | 16.7 |
| Degree | Intermediate | 94 | 20.5 |
|  | Bachelor | 107 | 23.4 |
|  | Master | 187 | 40.7 |
|  | PhD | 18 | 3.9 |
|  | Any other | 53 | 11.5 |
